# Optimization and validation of a fat-on-a-chip model for non-invasive therapeutic drug discovery

**DOI:** 10.3389/fbioe.2024.1404327

**Published:** 2024-06-25

**Authors:** Lindsey K. Huff, Charles M. Amurgis, Lauren E. Kokai, Rosalyn D. Abbott

**Affiliations:** ^1^ Department of Biomedical Engineering, Carnegie Mellon University, Pittsburgh, PA, United States; ^2^ Department of Bioengineering, University of Pittsburgh, Pittsburgh, PA, United States; ^3^ Department of Plastic Surgery, University of Pittsburgh, Pittsburgh, PA, United States

**Keywords:** adipose tissue, organ-on-a-chip, microfluidics, non-invasive monitoring, lipid metabolism

## Abstract

Obesity is a significant public health concern that is closely associated with various comorbidities such as heart disease, stroke, type II diabetes (T2D), and certain cancers. Due to the central role of adipose tissue in many disease etiologies and the pervasive nature in the body, engineered adipose tissue models are essential for drug discovery and studying disease progression. This study validates a fat-on-a-chip (FOAC) model derived from primary mature adipocytes. Our FOAC model uses a Micronit perfusion device and introduces a novel approach for collecting continuous data by using two non-invasive readout techniques, resazurin and glucose uptake. The Micronit platform proved to be a reproducible model that can effectively maintain adipocyte viability, metabolic activity, and basic functionality, and is capable of mimicking physiologically relevant responses such as adipocyte hypertrophy and insulin-mediated glucose uptake. Importantly, we demonstrate that adipocyte size is highly dependent on extracellular matrix properties, as adipocytes derived from different patients with variable starting lipid areas equilibrate to the same size in the hyaluronic acid hydrogel. This model can be used to study T2D and monitor adipocyte responses to insulin for longitudinally tracking therapeutic efficacy of novel drugs or drug combinations.

## 1 Introduction

Obesity is a chronic disease that accounts for approximately 2.8 million deaths and $173 billion dollars in medical costs annually according to the Centers for Disease Control and Prevention. The world health association defines obesity as abnormal fat accumulation that is a risk to the patient’s health. The weight of adipose tissue can range from 15%–30% of the body weight of healthy adults to as high as 50% of the body weight of morbidly obese adults (Loskill et al., 2017). Adipose tissue is a central metabolic organ responsible for regulating systemic energy homeostasis as a lipid reservoir and is located throughout the body as subcutaneous and visceral fat (Choe, 2016). Heart disease, stroke, type II diabetes (T2D), and certain cancers are comorbidities closely associated with obesity and pose serious public health threats to over 40% of the US population (Lim and Boster, 2023). T2D is a progressive disorder resulting from insulin resistance due to an excess amount of white adipose tissue (WAT) (Mileti, 2021) and is the most prominent comorbidity of obesity that is projected to affect more than 300 million people by 2025 (Longo, 2019). Due to the central role of adipose tissue in many disease etiologies and the pervasive nature in the body, engineered adipose tissues are essential for drug discovery, studying disease progression, and personalized medicine.

Many different adipose tissue models exist to represent adipose tissue *ex vivo.* One consideration is the culture of cells in two-dimensional (2D) or three-dimensional (3D) environments. Unlike 2D environments, 3D environments mimic the structure of the extracellular matrix and promote normal physiological function (Loskill et al., 2017; Wolff et al., 2022). Also, due to the buoyant nature of mature adipocytes, 3D environments contain the adipocytes and prevent detachment. Another consideration for these models is cell composition. General models consist of animal cell lines (Choi et al., 2020), human adipose derived stem cells (hASCs) (Bellas, Marra, and Kaplan, 2013; Abbott et al., 2015; Bender et al., 2020), mature adipocytes (DeBari et al., 2021), and explanted tissue seeded into scaffolds (Abbott et al., 2016; Abbott et al., 2018). Using stem cell lines is advantageous in that it allows for repeatable and reproducible methods, but often does not display the same phenotype and function as mature adipocytes. Animal cell lines do not recapitulate human physiology due to the species variation (Klingelhutz et al., 2018). For example, murine cell lines such as 3T3-L1 display a multilocular phenotype and secrete 1%–2% of the leptin of mature adipocytes (MacDougald et al., 1995). hASCs require lengthy differentiation times and in the absence of perfusion contain a multilocular phenotype (Gerlach et al., 2012; Abbott et al., 2015). Therefore, there is a critical need to develop a pre-clinical model that closely mimics the physiology of adipose tissue.

Organ-on-a-chip (OOC) models are microphysiological systems used to emulate the natural physiology of the human body by recreating the mechanical forces experienced by cells and the role of vasculature (nutrient supply and waste byproduct removal) through a perfusion system. Perfusion increases the nutrient supply, enriches the oxygen supply, and provides shear stress to the cells. OOC platforms can tailor the flow rate provided to the different cell types. For example, endothelial cells can be provided with high shear stress for proper endothelial maturation and functionality (Ballermann et al., 1998) while shielding adipocytes from high shear stress that compromises their viability (Daya, Loughlin, and Macqueen, 2007; Clark et al., 2009; Tanataweethum et al., 2018).

Various studies conducted recently have made significant strides in developing MPS to model adipose tissue *in vitro*. Recently, Tanataweethum et al. developed a microfluidic chip using adipocytes to fabricate an obese adipose tissue model. Through the induction of insulin resistance, they were able to develop a platform characterized by a decrease in Akt phosphorylation and Glut4 expression which could be used to further the understanding of signaling pathways and cellular mechanisms related to obesity on a 3D microscale (Tanataweethum et al., 2021). Further experiments were conducted to establish if the reversal of insulin resistance was possible with the introduction of rosiglitazone. In a similar study, Zhu et al. utilized adipose-derived stem cells to create a 3D adipose tissue model with hydrogel scaffolds (Rogal et al., 2022). By incorporating other cell types, such as fibroblasts and immune cells, they investigated the role of stromal cells in adipose tissue development and inflammation. This co-culture system allowed for the study of adipocyte behavior in the presence of surrounding cell types, mimicking the complexity of adipose tissue *in vivo*. Moreover, Li et al. developed an MPS using mature adipocytes to investigate adipose tissue metabolism and drug response (Li et al., 2017). Their platform allowed for long-term culture of mature adipocytes, enabling the study of adipocyte viability and functionality over an extended period. This system provided valuable data on drug-mediated effects on adipocyte glucose uptake and lipid metabolism. While the aforementioned platforms have advanced the field of modeling adipose tissue *in vitro* on a microfluidic platform, there is still much to explore and understand. For example, MPS predominantly relies on gathering terminal endpoint analysis rather than continuous data over time, hindering the exploration of dynamic changes and responses occurring within the model. Therefore, the pursuit of non-invasive analyses are necessary to observe the multifaceted nature of adipose tissue more accurately and further advance our understanding of its role in health and disease.

**FIGURE 1:**
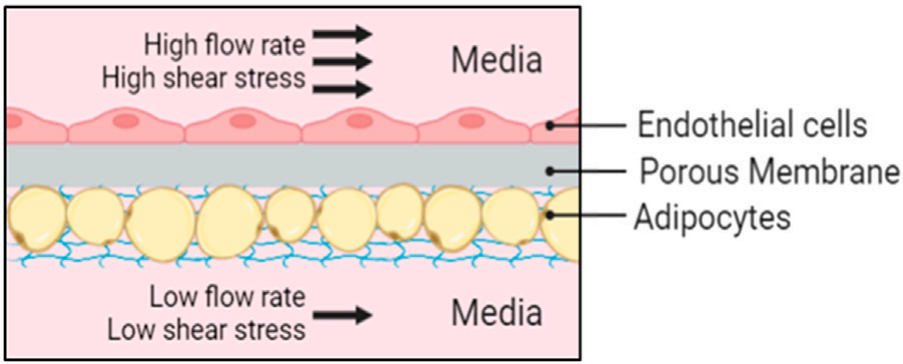
Schematic representation of the Micronit device. Endothelial cells are cultured in-line to the fluid flow with high shear stress and adipocytes are protected from high flow rates in an in-set chamber with nutrient supply provided from diffusion below at a low flow rate.

In this paper, we construct an adipose tissue depot in a Micronit OOC platform (Figure 1). The Micronit device creates a 3D microenvironment that has two distinct compartments, separated by a permeable membrane, that allows adipocytes to be entrapped in the bottom region of the device, shielding the cells from high flow rates, and keeping the buoyant cells submerged in the system. The scale of the perfusion compartment (millimeters) minimizes the obstruction of flow from hydrophobic secretions, an advantage over other approaches on 2D microfluidic devices. Benefits of this platform also include dynamic fluid flow between chambers that allows crosstalk between cell types.

Accounting for patient-to-patient variability by including primary adipocytes is another important consideration for creating a tissue engineered model that has human physiological relevance. Patient demographics such as age, gender, ethnicity, and medical history affect the phenotype and behavior of adipose tissue (DeBari et al., 2023). For example, an increase in age alters adipose tissue distribution (relative loss in peripheral subcutaneous fat), function, and impacts adipokine synthesis ultimately resulting in a chronic state of low-grade systemic inflammation (Mancuso, 2019). Adipose tissue remodeling can be influenced by sexual dimorphism due to the differences in sex hormones between genders (Moreira-Pais et al., 2020) and influenced by a patient’s medical history including weight fluctuation. Furthermore, the pharmacokinetics of drugs vary with patient demographics and obesity state (Cheymol, 2012). Current models use cell lines and thus limit our knowledge of adipose tissue behavior to one group of people. Therefore, an adipose tissue model is needed that accounts for patient-to-patient variability (Abbott et al., 2018).

**FIGURE 2:**
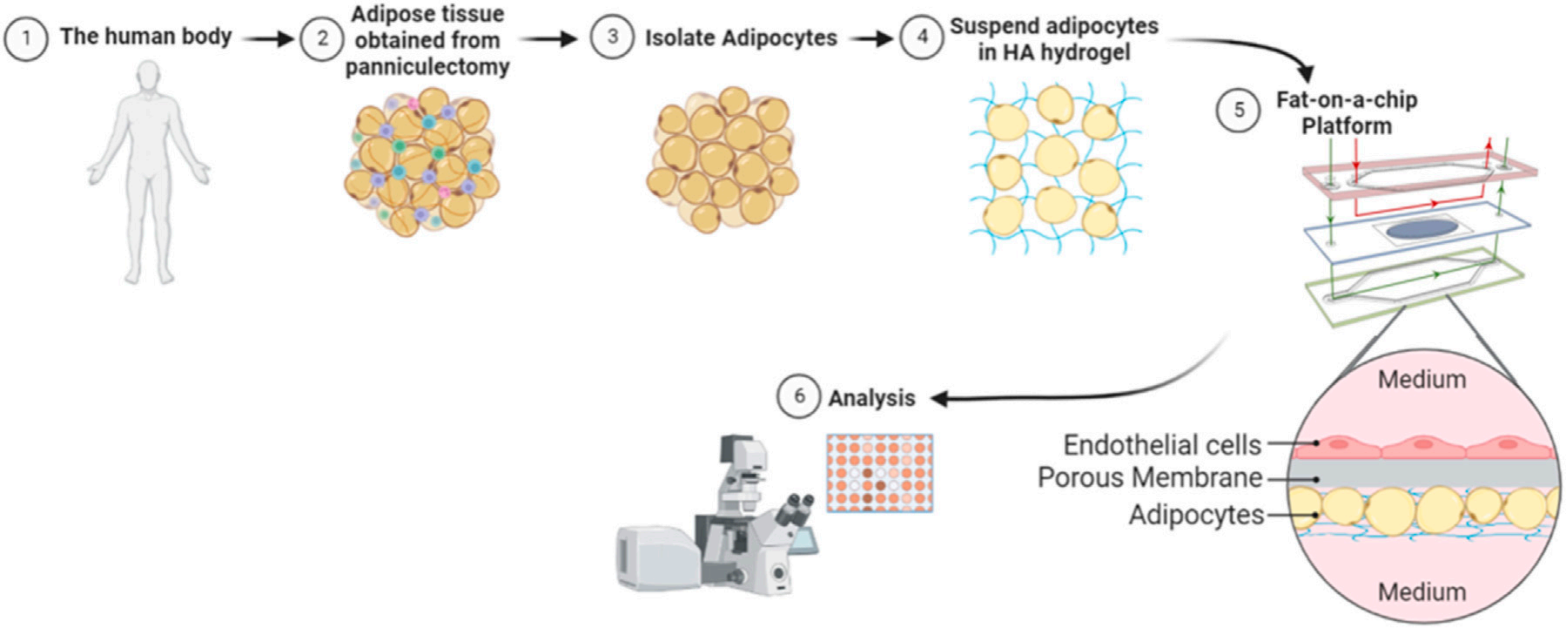
Illustration of methodology. Whole adipose tissue was explanted and processed for adipocyte isolation. The isolated mature adipocytes were mixed with a hyaluronic acid hydrogel and seeded onto each Micronit chip. Separated by a permeable membrane, endothelial cells were seeded in an adjacent chamber to the adipocytes. Perfusion on the top and bottom of the device provided high shear stress to endothelial cells and low shear stress to adipocytes. The chips were cultured on the Micronit platform and then underwent further analysis.

In this paper, we develop a FOAC model with human primary adipocytes (Figure 2). Specifically, we assessed the reproducibility, viability, growth, and insulin response of adipocytes in a Micronit platform while using resazurin as a non-toxic and non-invasive functionality/viability marker. Our FOAC model uses a commercially available Micronit perfusion device and can be used to monitor adipocyte responses to insulin for longitudinally tracking drug efficacy and assessing therapeutic efficacy of novel drugs.

## 2 Materials and methods

### 2.1 Device assembly

All the microfluidic device parts were obtained from Micronit (Enschede, Netherlands) unless otherwise noted. The Micronit chip holder was sterilized by spraying with 70% EtOH and aspirating off the alcohol inside the laminar flow hood. Once the chip holder was fully dried and opened, the autoclaved ferrules were placed into inlet (2 and 5) and outlet (8 and 11) ports of all four chip frames with sterile forceps. When all 16 ferrules were firmly in place, the three glass layers were placed into the frames with forceps in the following order: 1) bottom layer (gasket facing up), 2) dummy layer, and 3) top layer (gasket facing down). After visual inspection for ferrule or chip layer misalignment, the chip holder was closed and locked into position for priming. Tubing for both influx and efflux was constructed from three separate components: 1) IDEX Peek main tubing (11–16 cm influx, 22 cm efflux, red) (Optimize Technologies, Oregon City, OR), 2) Masterflex C-flex connector tubing (1.5 cm, clear) (IDEX, Lake Forest, IL), and 3) TBG NAT Peek tubing inserts (1.5 cm, tan) (Avantor, Gelsenkirchen, Germany). Prior to device assembly, each tubing component is cut with a straight razor, autoclaved, and connected. The tubing was inserted, TBG NAT PEEK side first, into its corresponding inlet (2 and 5) and (8 and 11) and outlet ports for all four chips frames within the holder. Once the tubing was properly inserted, eight 10 mL syringes were filled with Dulbecco’s Modified Eagle Medium (DMEM) (Gibco, Grand Island, NY) and loaded onto the PHD Ultra Syringe Pump (Harvard Apparatus, Holliston, MA). A 20-gauge, 0.5-inch blunt needle was screwed onto each syringe and connected to the corresponding inlet tubing. After, outlet tubing was placed into the top of outlet conical tubes for effluent collection. Following tubing insertion, the syringe pump was activated, and the system was primed at a flow rate of 1 mL/min until media was perfused throughout the system. If done correctly, bubbles will be absent in the window of the chip frames (if bubbles were present the process was repeated until the system was primed correctly). Once primed, the inlet and outlet tubing were disconnected from the syringes and conical tubes, respectively. The chip holder was opened, and the dummy intermediate layers were replaced with the pre-seeded adipocyte/endothelial intermediate layers. Subsequently, the chip holder was closed and locked into place for cellular perfusion. The inlet and outlet tubing were reinserted into the chip. The syringes and conical tubes were discarded and replaced before the inlet and outlet tubing was reinserted into the holder. The replacement syringes were loaded into the pump, which was reprogrammed to perfuse at 30 μL/h. Following assembly, the system was placed into a 37°C incubator for culturing.

### 2.2 Chip synthesis

During the initial FOAC optimization trials, the standard intermediate chip membrane was used to seed our adipocyte-hystem mixture on-chip. Manufactured by Micronit, the intermediate layer of the chip is comprised of a 3-micron pore, PET membrane that allows for bidirectional media perfusion. However, unlike the top and bottom chip layer, which can be sterilized and reused in perpetuity, the adipocyte-seeded membrane on the intermediate layer must be removed and disposed of at the end of each trial. To achieve a greater level of reproducibility, a system of membrane replacement was developed, which allowed for multiple uses of each intermediate chip through the adherence of a sterile membrane (Sterlitech, Auburn, WA) prior to each experiment. Following sample collection at the end of perfusion, the used membrane on the intermediate layer was carefully removed with forceps and discarded. The intermediate layer was then washed with soap and water to remove remnant media and placed in 70% EtOH before being autoclaved. Using a surgical scalpel, the new membranes were cut into 20 × 16 mm rectangles from a 47 mm diameter sheet, under sterile conditions. Subsequently, a UV-sensitive NOA72 liquid adhesive (Edmund Optics, Barrington, NJ) was dispensed onto the sterile intermediate glass layer via a 3 mL syringe. The rectangular membrane was then placed onto the adhesive and was exposed to ultraviolet light for 1 h. Once cured, the intermediate layer was used for cell-seeding.

### 2.3 Isolation of preadipocytes

Subcutaneous adipose tissue was obtained from elective abdominoplasty and abdominal panniculectomy procedures at the University of Pittsburgh Medical Center (UPMC) with approval by the University of Pittsburgh Institutional Review Board (IRB No. 0511186) and informed consent. The tissue was processed the day of the surgery. Blunt dissection was used to separate the adipose tissue from the skin followed by pulse blending to break up the tissue. An equal volume of liquified adipose tissue and warmed sterile DPBS (Thermo Fisher Scientific, Waltham, MA) was used to wash the tissue until the PBS remained clear after washing. An equal volume of warmed collagenase solution (1% BSA (Sigma-Aldrich, St. Louis, MO) and 0.1% collagenase type I (Gibco, Grand Island, NY) in PBS) was then added to the washed adipose tissue and incubated for 1 h at 37°C, and 5% CO_2_. After incubation, the solution was centrifuged at 300 *g* for 5 min at room temperature, separating the solution by oil, primary adipocytes, collagenase solution, and stromal vascular fraction (pelleted), from top to bottom. The oil was aspirated and the primary adipocytes were transferred onto a 1 mm sieve, followed by a 350 µm sieve, using warmed PBS to filter the primary adipocytes from the other non-cellular debris. The filtered primary adipocytes were centrifuged at 300 *g* for 5 min at room temperature, and again the solution was separated by oil, primary adipocytes, and PBS, from top to bottom. The oil was aspirated and the primary adipocytes were transferred into a separate tube.

### 2.4 Human umbilical vein endothelial cell (HUVEC) seeding

To prepare the chip for endothelial cell seeding, a 10 μg/mL fibronectin in PBS solution was prepared. 100 μL of the fibronectin solution was dispensed uniformly onto the membrane of each intermediate chip layer within a laminar flow hood. After coating thoroughly, the intermediate chip layers were placed in a 37°C incubator for 24 h until the solution evaporated. Cryopreserved, second passage human umbilical vein endothelial cells (HUVECs) were thawed and expanded with EGM-2MV (Lonza, Basel, Switzerland) in a T-75 flask until confluent. After confluence was achieved, the HUVECs were lifted with 0.05% Trypsin-EDTA (Thermo Fisher Scientific, Waltham, MA), resuspended to a density of 1 × 10^6^ cells/mL, and seeded uniformly onto the top side of the membrane with a volume of 100 μL. Subsequently, the chips were cultured statically for 3 days in a 1:1 mixture of DMEM and EGM-2MV to allow for expansion.

### 2.5 Hydrogel mixture and adipocyte cell seeding

The HyStem hyaluronic acid (HA) hydrogel (Advanced Biomatrix, Carlsbad, CA) was made using a 4:1 ratio of Thiol-modified hyaluronan (Gycosil) and Thiol-reactive PEGDA crosslinker (Extralink). Gelation occurs within 20 minutes of all components being added together. After a 10-min gel time, an equal volume of HA hydrogel and primary adipocytes were mixed. 35 μL of the mixture was seeded onto each chip and allowed to gel for an additional hour in the incubator. Media was added and the chip was incubated overnight. The chips were then transferred onto the Micronit platform.

### 2.6 Culture and perfusion

The Micronit system was set up with a syringe pump to provide constant perfusion of DMEM supplemented with 10% Fetal Bovine Serum (FBS) (Gibco, Grand Island, NY) and 1% Penicillin Streptomycin (P/S) (Gibco, Grand Island, NY) to the chips. Tubes were connected to the syringes to supply media to the top chamber of the chip (endothelial cells) and bottom chamber of the chip (adipocytes) at a flow rate of 100 μL/h and 30 μL/h, respectively. Tubes were connected to the outlet 15 mL conical tubes to collect the waste which was used for various assays throughout the culture. The outlet flow was collected and frozen in a −80°C freezer every 24 h for use in future assays. Additionally, syringes were changed every 24 h under sterile conditions to ensure fresh media was perfusing the system. The Micronit perfusion system was used to culture the chips for four to 10 days and then processed for immunofluorescence staining.

### 2.7 Resazurin

Powdered resazurin (Thermo Fisher Scientific, Waltham, MA) was reconstituted to a 1 mM resazurin solution with PBS and then further diluted with DMEM to make a 0.1 mM resazurin working concentration prior to perfusion. 10 mL syringes containing 3 mL of the resazurin working concentration were used to perfuse the Micronit chips. Media was collected every 24 h for a 10-day period under sterile conditions. Media changes occurred every 48 h. In this process, the syringes were discarded and replaced with new 10 mL syringes containing 3 mL of a freshly made 0.1 mM resazurin in DMEM solution. To prevent fluorophore degradation, all media perfusion, collections, and changes were performed away from direct light exposure. When necessary, tin foil was used to cover the syringes containing resazurin. To compensate for temperature and pH-induced interferences in resazurin signaling from the incubator conditions (37C, 5% CO2), a separate conical tube containing 0.1 mM resazurin working concentration was perfused through one chip in the Micronit system, to serve as a negative control. Thus, each experiment consisted of 1 control chip and 3 experimental chips, and each experiment was repeated 3 times to assess the variability of different patients. The plate reader was used to measure the absorbance at 560/590 nm. Metabolic activity was measured every other day of culture until trial completion.

For initial resazurin measurements, the volume of adipocyte hydrogel mixture on three chips were 20 μL, 30 μL, and 40 µL. The chips ran for 120 h and resazurin was measured at 24 h and 120 h. Following resazurin measurements, the volume of adipocyte hydrogel mixture was constant and resazurin was measured every other day.

### 2.8 Immunostaining

Viability was assessed using LIVE/DEAD Assay kits (Invitrogen, Waltham, MA) throughout the experiment. Samples were washed three times with warmed PBS for 5 min in the incubator and then stained with calcein-AM (1:2000) and Ethidium homodimer-1 (1:500) for 30 min in the incubator. The stained samples were imaged using a Zeiss LSM 700 Confocal microscope using signals (excitation/emission) from calcein-AM (517 nm/494 nm) with the 488 nm laser and ethidium homodimer-1 (617 nm/528 nm) with the 555 nm laser, and given pseudocolors Green and Red, respectively. As there was autofluorescence in the red channel, a threshold was determined against the PEG membrane on the chip.

Cellular morphology was assessed in samples that were washed three times with warmed PBS for 5 min in the incubator and then fixed with 10% neutral buffered formalin for 30 min at room temperature. Samples were then washed three more times with PBS for 5 min 0.1% Triton-X100 was added to the samples for 15 min at room temperature, followed by one PBS wash for 5 min. The samples were stained with BODIPY (1:4000) (Invitrogen, Waltham, MA), DAPI (1:400) (Sigma-Aldrich, St. Louis, MO) and Alexa flour phalloidin 555 (1:400) (Thermo Fisher Scientific, Waltham, MA) for 1 h and washed three times with PBS for 5 min. The stained samples were imaged using a Zeiss LSM 700 Confocal microscope using signals from BODIPY (493 nm/503 nm) with the 488 nm laser, DAPI (358 nm/420–440 nm) with the 405 nm laser, and Alexa flour phalloidin 555 (555 nm/565 nm) with the 555 nm laser. These signals were given the pseudocolors green, blue, and red, respectively. Z-stack images were obtained from each sample and maximum intensity projections were used to create a combined image. ImageJ Software was used to analyze images and quantify adipocyte size.

### 2.9 Triglyceride and DNA content

Samples were collected from tissues following adipocyte isolation methods, lysed in TE buffer, and stored at −20 C until the assays were performed. Triglyceride and DNA content was assessed using thawed samples with the EnzyChrom Triglyceride Assay Kit (BioAssay Systems, Hayward, CA) and Quant-iT Picogreen DNA Assay Kit (Invitrogen, Waltham, MA), respectively following the manufacturer’s procedures.

### 2.10 Glucose uptake

For glucose uptake experiments, samples were perfused with 100 nM insulin (Gibco, Grand Island, NY) dissolved in 1% acetic acid and diluted in low glucose DMEM. Media changes occurred every 48 h under sterile conditions. Effluent media was collected every 24 h and stored at −80°C until the conclusion of the experiment. Samples were thawed at room temperature prior to glucose concentration measurements. Glucose concentration was determined using the GlucCell™ Glucose Monitoring System (Thermo Fisher Scientific, Waltham, MA). Effluent samples were diluted with standard DMEM by a factor of two to be within the optimal glucose monitoring range. Following device calibration for DMEM media, 2 µL of the diluted sample was dispensed onto a sterile Petri dish. Subsequently, a GlucCell™ Glucose Test Strip was inserted into the glucose monitoring device and placed in the 2 µL sample for 10 seconds, to ensure accuracy. This process was repeated in duplicate for each chip at every 24-h collection interval. Each experiment consisted of 2 chips without insulin supplemented, 2 chips with insulin, and 2 control groups with no cells, and each experiment was repeated 3 times to assess the variability of different patients. The glucose uptake was calculated from the difference in glucose levels between the control groups and the chips.

### 2.11 LDH secretion

Cytotoxicity was evaluated by measuring the Lactate Dehydrogenase (LDH) concentration of the effluent media samples at 48-h intervals for 20 μL, 30 μL, and 40 µL mixtures. Perfused media was collected and stored at −80 C until the completion of the experiment at 240 h, resulting in five timepoints for data collection. Subsequently, samples were thawed at room temperature and measured using an LDH ELISA Assay Kit (LS Bio, Shirley, MA). Fluorescence detection was read with a plate reader.

### 2.12 Statistics

ImageJ software was used to quantify the lipid diameters. GraphPad Prism software was then used for all statistical analyses. A Student’s t-test was performed for data comparing two groups while a one-way ANOVA was performed with a Tukey’s *post hoc* multiple comparison test for experiments with more than two groups. For all statistical analyses, significance was defined as *p* < 0.05. Additionally, a two-way ANOVA was performed with a Tukey’s *post hoc* multiple comparison test for experiments comparing two groups to two variables.

## 3 Results and discussion

### 3.1 Initial patient variability in human adipose tissue samples

**FIGURE 3:**
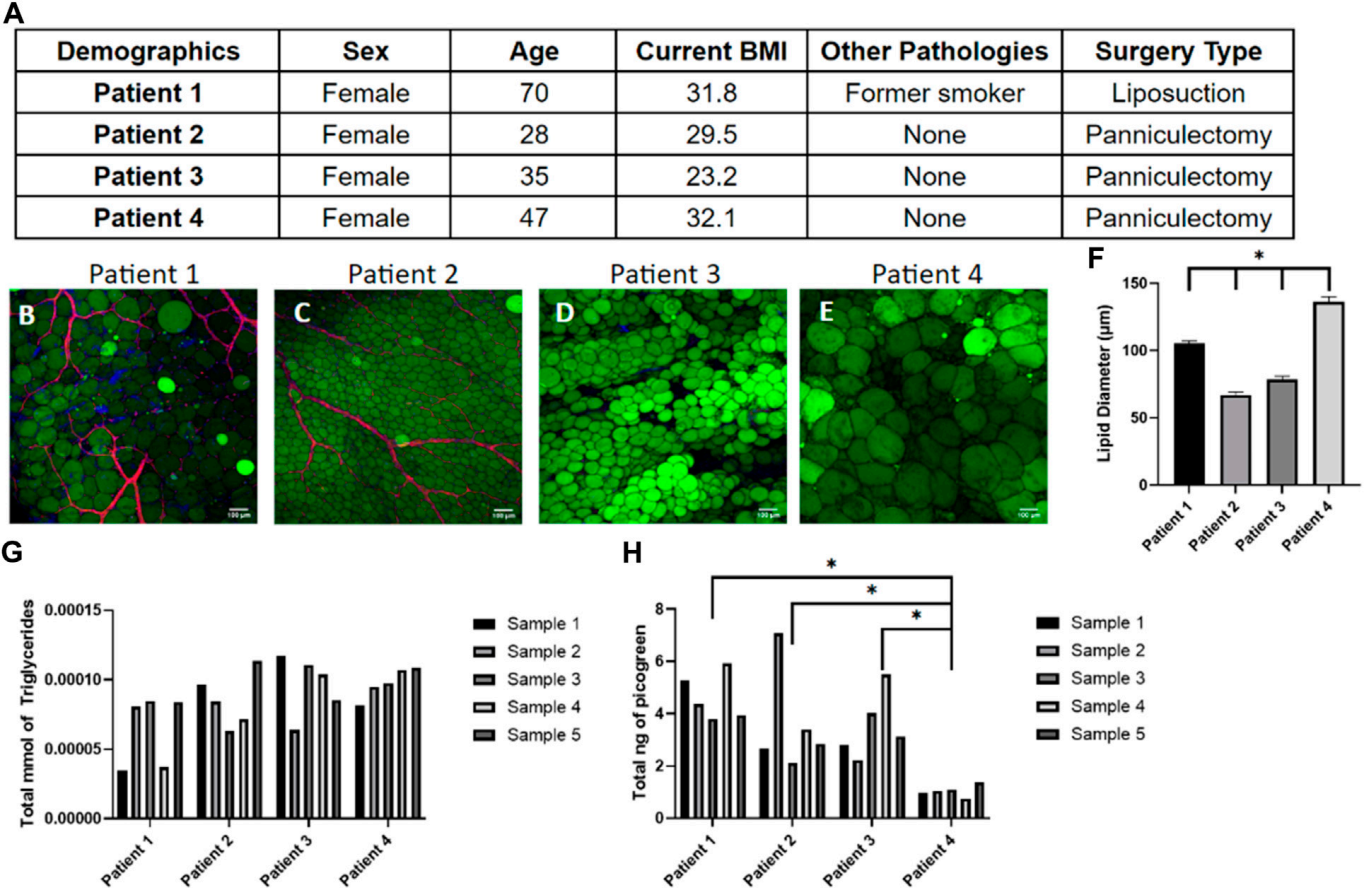
Tissue samples display high variability in adipocyte size, triglyceride content, and number of cells within patients and between patients. **(A)** Demographics of the four patient samples. **(B–E)** Day 0 images of adipose tissue were stained with BODIPY (green = lipids), DAPI (blue = nuclei), and Phalloidin 555 (red = actin cytoskeleton) from **(B)** patient 1, **(C)** patient 2, **(D)** patient 3, and **(E)** patient 4 demonstrating the large variability in adipocyte sizes and amount of vasculature between samples. Scale bar = 100 μm. Images were quantified for **(F)** lipid diameters at day 0. A one-way ANOVA with a Tukey’s post hoc test were performed on the mean lipid diameter of each patient and results show statistically significant differences in lipid diameter between patients 1–4 (*p* < 0.05). Error bars represent the standard error of the mean. **(G)** Triglyceride and **(H)** DNA content varied within patient samples and between patient samples, underscoring the need to normalize data for each device separately (*n* = 5). For statistical analyses of triglyceride and DNA content, a one-way ANOVA and post hoc Tukey’s test were performed on the mean of each patient and results show statistically significant differences in DNA content between patient 4 and patient 1–3 (*p* < 0.05). *n* = 4.

While it is widely accepted that obesity is linked with adipose tissue dysfunction due to the chronic inflammation of adipose tissue (Fuster et al., 2016; Quail and Dannenberg, 2019; Koenen et al., 2021), obesity is not the only factor that alters the functionality and metabolic activity of adipose tissues. Patient demographics such as age, gender, ethnicity, life-style choices, and medical history affect tissue function (DeBari et al., 2023) which contribute to the high variability in adipose tissue models constructed from primary human samples (Abbott et al., 2018). In this study, patient demographics were catalogued (Figure 3A) and compared to the quantitative data of tissue samples to show the patient-to-patient variability of patient samples. As expected, there is large variability in adipocyte size and amount of vasculature in the starting samples (Figures 3B–E). Generally, a higher BMI is associated with larger adipocyte sizes though this relationship is nonlinear once reaching the obese range (Laforest et al., 2015). This is somewhat consistent with the data collected from the four patients, as patient 4 has the largest BMI and largest adipocytes, however patient 3 has the lowest BMI but not the smallest (Figure 3F). Given the large variability within and between patients histologically, we next sought to quantify the variability. As expected, the triglyceride (Figure 3G) and DNA content (Figure 3H) varied within the same sample and between different patient samples. This variability emphasizes the importance of developing a consistent seeding method of primary cells and monitoring data points for every chip individually to account for the noise between and within human samples.

### 3.2 Reproducibility of 3D culture conditions

**FIGURE 4:**
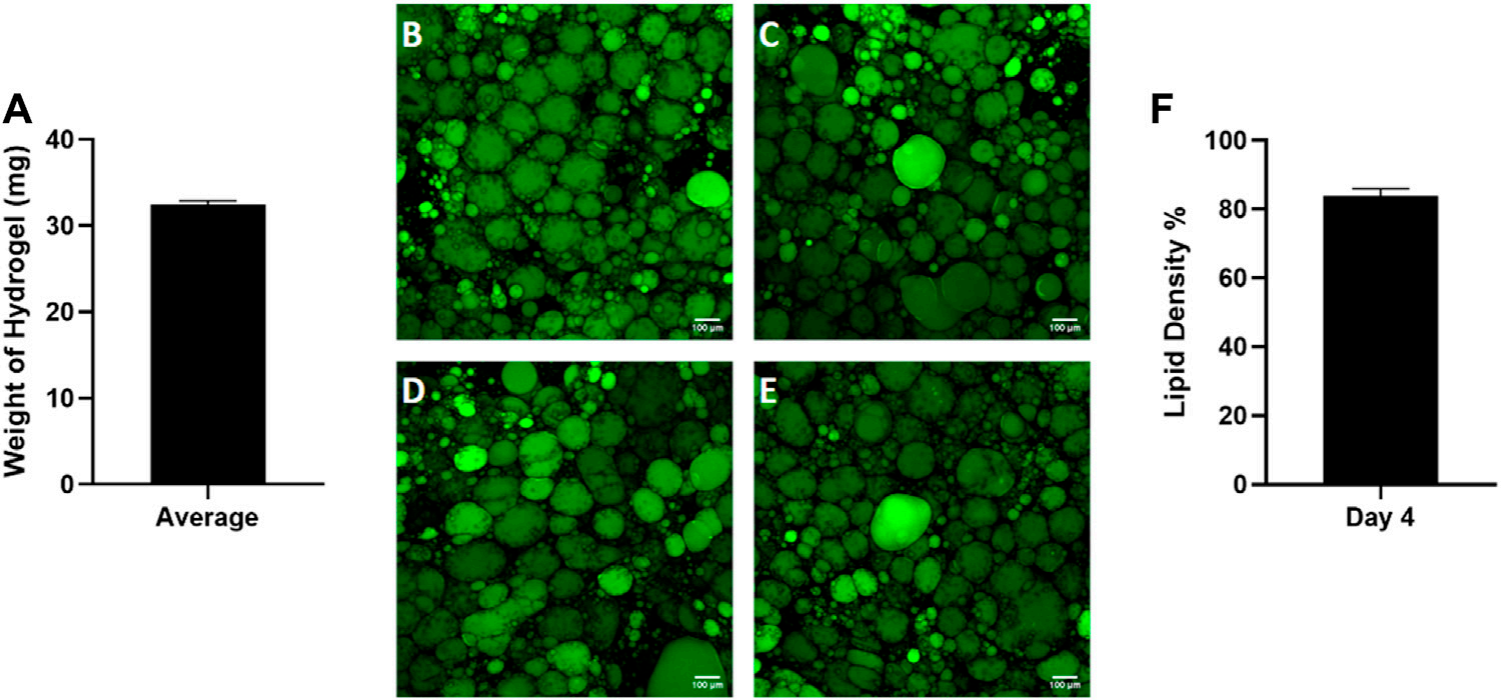
Consistent adipocyte seeding in the hyaluronic acid hydrogel on the Micronit device. Day 4 images of hydrogels from the same patient on **(A)** chip 1, **(B)** chip 2, **(C)** chip 3, and **(D)** chip 4. Day 4 images of adipose tissue were stained with BODIPY (green = lipids), DAPI (blue = nuclei), and Phalloidin 555 (red = actin cytoskeleton). The low standard error of the mean (error bars) for the **(E)** weight of hydrogel on each chip and **(F)** lipid density (surface area coverage) indicates a consistent seeding. Scale bar = 100 μm. *n* = 4.

Given the high variability in starting tissue, we sought to assess the reproducibility of reconstituting primary adipocytes in the hyaluronic acid hydrogel mixture. Preliminary data (Supplementary Figure S1) was performed to optimize the hydrogel ratio to 4:1. After combining the hydrogel with adipocytes, the hydrogel mixture weight was recorded by calculating the difference between the weight of the microfluidic chip before and after adding the hydrogel mixture (Figure 4A). The consistent weight of the hydrogel indicates homogenous hydrogel seeding despite the buoyant nature of adipocytes. After culture, the lipid density of cells on each chip was assessed (Figures 4B–E) demonstrating consistency as well (Figure 4F). This indicates the hydrogel seeding method creates a uniform adipocyte seeding on chip that is reproducible amongst chips.

**FIGURE 5:**
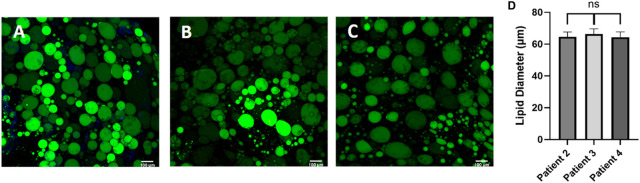
Post processing images of adipocytes in the hyaluronic acid hydrogel show consistent lipid diameters, regardless of patient demographic variability. Day 0 post processing images of adipocytes suspended in HA hydrogel were stained with BODIPY (green = lipids), (DAPI = nuclei), and Phalloidin 555 (red = actin cytoskeleton) from **(A)** patient 2, **(B)** patient 3, and **(C)** patient 4. Scale bar = 100 μm. Images were quantified for **(D)** lipid diameters. A one-way ANOVA with a Tukey’s *post hoc* test was performed on the mean lipid diameter of each patient and results show no statistically significant differences in lipid diameter between patients (*p* < 0.05). Error bars represent the standard error of the mean. *n* = 3.

Next, we sought to evaluate the consistency of adipocyte sizes in our platform. Interestingly, once the adipocytes were seeded in a homogenous hydrogel their unilocular lipid diameter was consistent across patients (Figure 5) despite their initial starting variability (Figures 3B–F). Adipocyte cell size is known to vary with extracellular matrix composition where enhanced collagen deposition restricts adipocyte size (Dalmas et al., 2015). Our findings support the concept that adipocyte size is highly dependent on extracellular matrix stiffness, as cells adapted rapidly to their new hydrogel environment. This result also supports the reproducibility of our platform for culturing human primary adipocytes.

### 3.3 Viability assessment in 3D culture

**FIGURE 6:**
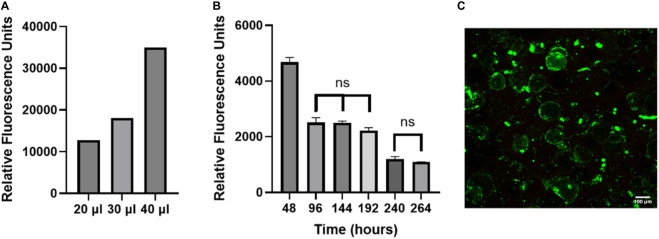
The non-invasive resazurin assay can be used on-chip to evaluate cellular viability. **(A)** The volume of adipocyte hydrogel mixture on three chips was increased by an increment of 10 μl, starting at 20 μl. Resazurin was used to measure the metabolic activity of the adipocytes by reading the relative fluorescence at 24 h. The data displays proportional increases in the relative fluorescence for each chip of varying volume, verifying that resazurin can be used with the FOAC system. **(B)** Resazurin data during 7-day trial. For the statistical analysis, a one-way ANOVA and post hoc Tukey’s test were performed (*p* < 0.05). Finally, live/dead images were performed on the final day of cultures for **(C)** resazurin experiment with 95% viability. Scale bar = 100 μm. n = 3.

Viability of cells on OOC models is generally assessed using Live/Dead staining (Khalid et al., 2020; Yang et al., 2021; Kim et al., 2022; Shao et al., 2023), however this is a destructive endpoint cell assay and only provides information at one time point. Due to human variability, the cellular function and viability needs to be assessed for every chip. Using a non-invasive readout to assess cellular function and viability will allow data to be collected from every chip on the system. Resazurin is a non-toxic soluble dye that is reduced to a highly fluorescent form (resorufin) in proportion to the amount of metabolically active cells (Uzarski et al., 2017). While resazurin has been routinely used in static culture (allowing ample time to reduce resazurin), we wanted to test the accuracy of using this assay in our FOAC culture which was undergoing constant perfusion (at a slow rate). To validate that resazurin can be used on a perfusion device, the number of cells was incrementally increased to evaluate the sensitivity of the signal in the effluent. As the volume of adipocytes increases, the relative fluorescence signal from resorufin proportionally increased (Figure 6A). This experiment shows resazurin can be used as a live cell assay indicator in FOAC systems.

FOAC cultures with consistent numbers of cells per chip, were monitored with resazurin (Figure 6B). At 48 h, the relative fluorescence is significantly higher than the other time points, likely due to the stress experienced by the adipocytes during the isolation processing. Stress increases the function and size of mitochondria (Picard and McEwen, 2018). Between 96–192 h, there are no significant changes in the relative fluorescence, followed by a significant decrease at 240 h, where it then remains statistically unchanged to 264 h. The consistent cellular activity, after the initial drop at 48 h, indicates the cells remain viable throughout the experiment. An LDH readout was also performed on the system to measure cell death and further validate using resazurin as a marker for viability. The LDH samples were below the bounds of the assay kit indicating there was minimal cell death (Supplementary Figure S2). For further validation, the viability of the cells was quantified with LIVE/DEAD (Figure 6C). On the final day of culture, there is an average 95% adipocyte viability, confirming the use of resazurin on the Micronit platform as a marker of active, live cells.

**FIGURE 7:**
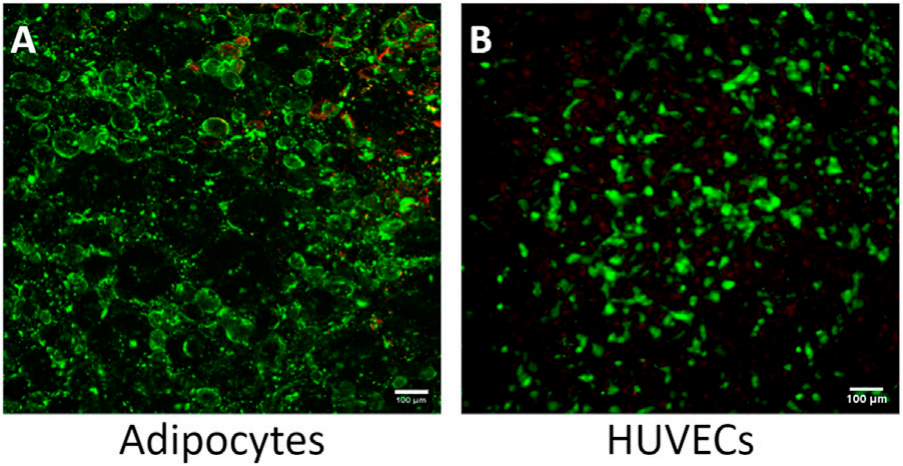
The Micronit device can support a viable co-culture of adipocytes and HUVECS. **(A)** Co-culture experiment of adipocytes with 90% viability and **(B)** co-culture experiment of HUVECS with 95% viability. Live cells stained green and dead nuclei are red. Scale bar = 100 μm. *n* = 2.

Although adipose tissue is highly vascularized, most adipose tissue models do not incorporate a vascular unit (Clark et al., 2009; Clark et al., 2010; Lai et al., 2012; Dugan et al., 2014; Godwin et al., 2015; Loskill et al., 2017; Tanataweethum et al., 2018). The high microvascular density in adipose tissue serves many functions, including providing nutrients and oxygen, removing metabolic products, delivering non-adipose-tissue-derived growth factors, cytokines, and hormones, and transporting adipose-tissue-derived endocrine signals to other organ systems (Cao, 2013). The microvasculature also provides paracrine interactions where VEGF-triggered signaling by endothelial cells induces preadipocyte differentiation (Fukumura et al., 2003) and preadipocytes further stimulate endothelial migration (Borges et al., 2007). Incorporating endothelial cells creates a selective endothelial barrier to control diffusion of nutrients and other compounds to the adipocyte compartment. Therefore, we evaluated the feasibility of co-culturing adipocytes and HUVECs in our platform. While adipose tissue endothelial cells are the native cell type, HUVECs are commonly used as a model system for studying endothelial cells in adipose tissue (Benmeridja et al., 2020; Zhu et al., 2022; Peirsman et al., 2023). The live/dead assay shows an average of 90% viability for both cell types (Figures 7A, B). Resazurin and LIVE/DEAD imaging data suggest that the cells remain viable during co-culture on the Micronit platform. These images were taken from the two distinct compartments in the Micronit platform, as seen from the schematics in Figures 1, 2. After confirming that adipocytes and HUVECs remain viable in co-culture the following experiments use an adipocyte-only model to further optimize the platform without confounding variables from endothelial cells.

### 3.4 The effects of 3D culture on adipocyte size

**FIGURE 8:**
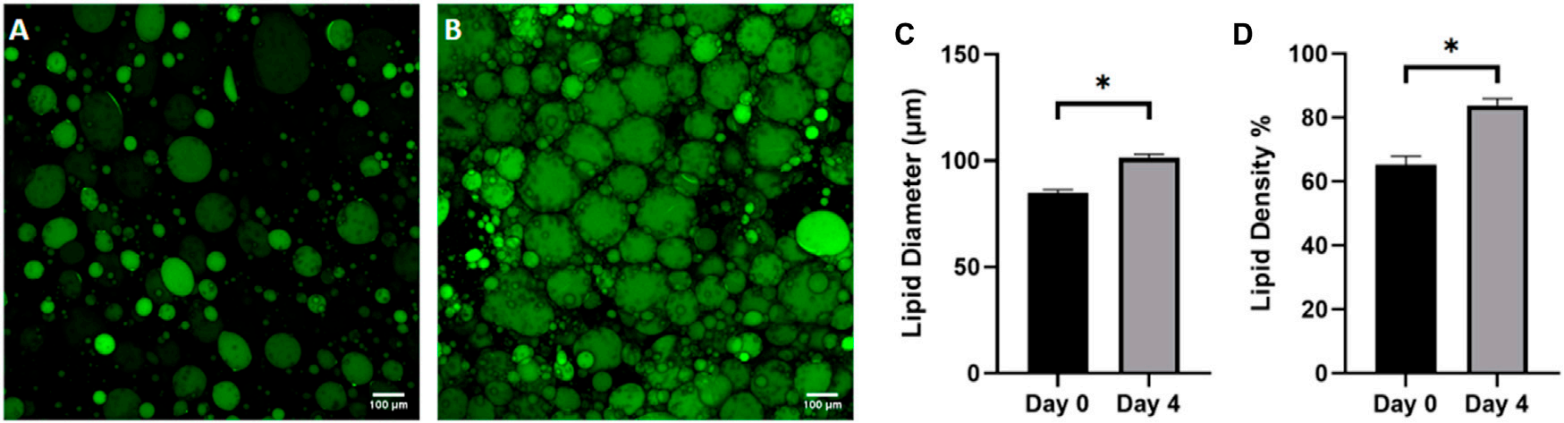
Adipocyte hypertrophy was observed on-chip after 4 days of high glucose supplementation. Representative images of **(A)** cells before supplementation (day 0) and **(B)** after 4 days in high glucose conditions (4.5 g/L), stained with BODIPY (green = lipids). Scale bar = 100 µm. Images were quantified for **(C)** lipid diameters and **(D)** percentage lipid density (surface area coverage) at day 0 and day 4. For statistical analyses, *t*-test were performed on the means and results show statistically significant differences in lipid diameter and lipid density between day 0 and day 4 (*p* < 0.05). Error bars represent the standard error of the mean. *n* = 3.

WAT is a highly dynamic tissue capable of morphological changes based on environmental nutrient signaling. Adipocyte size has been shown to fluctuate from 20–300 μm in diameter as a mechanism for accommodating nutrient uptake and depletion (Stenkula and Erlanson-Albertsson, 2018) For example, a short-term, overfeeding study by McLaughlin *et al* demonstrated that a 3 kg increase in body weight in obese individuals resulted in a 10% size increase in pre-hypertrophic adipocytes (McLaughlin et al., 2016). To assess the ability of adipocytes to dynamically regulate size based on nutrition on the FOAC platform, images were taken on day 0 (Figure 8A) and day 4 (Figure 8B), after constant perfusion of high-glucose (4.5 mg/mL) DMEM. A significant increase in adipocyte diameter is observed after the 4-day culture (Figures 8C, D). It is speculated that FBS plays a role in lipid metabolism, thus the increase in adipocyte size is the result of prolonged exposure to elevated levels of sugar and carbohydrates (Kersten, 2001) and indicates functional lipogenesis on the FOAC platform.

### 3.5 Adipocyte glucose response to insulin

Finally, we sought to evaluate the functionality of the adipose platform. Insulin plays a crucial role in regulating glucose homeostasis in adipocytes *in vivo* (Norton et al., 2022). Upon binding to its receptor, insulin triggers a cascade of intracellular signaling events that lead to the translocation of glucose transporter GLUT4 to the cell membrane, enabling glucose uptake into the adipocyte. This process is essential for maintaining glucose levels in the body and is a critical function of adipose tissue. Additionally, insulin inhibits lipolysis, the breakdown of stored triglycerides into free fatty acids, which helps prevent excessive release of fatty acids into the bloodstream and contributes to lower circulating glucose levels. Impaired insulin signaling and dysregulated adipocyte function are closely linked to the development of metabolic disorders such as obesity and type 2 diabetes (Norton et al., 2022). Given the pivotal role of insulin in adipocyte glucose uptake and its relevance in glucose homeostasis *in vivo*, the insulin-mediated glucose uptake response was selected to evaluate the functionality of the microfluidic platform. By measuring the glucose uptake in response to insulin, we aimed to demonstrate the platform’s ability to recapitulate an *in vivo* physiological response in a 3D *in vitro* environment.

**FIGURE 9:**
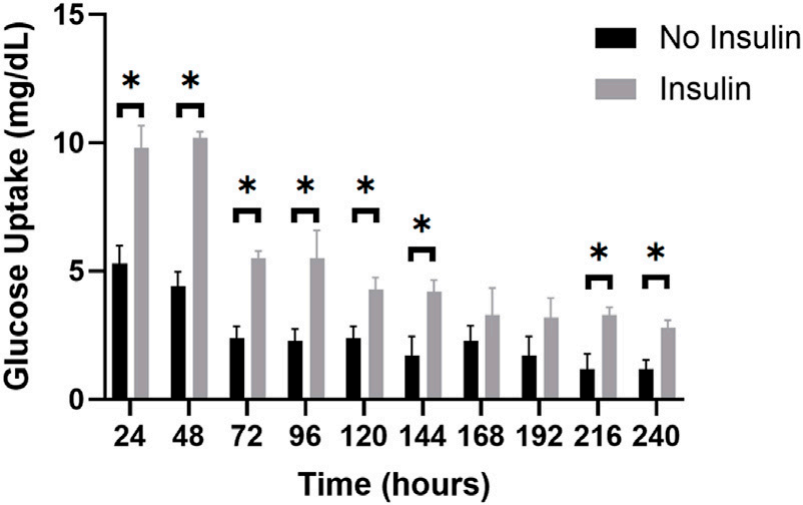
The average glucose uptake (mg/dL) of insulin-perfused adipocytes and non-insulin control adipocytes at 24-h timepoints over a 10-day trial. The graph reveals a clear trend in glucose uptake for both groups over the experimental period. Throughout the 10-day trial, the insulin-perfused adipocytes consistently exhibited a higher average glucose uptake compared to the non-insulin control group at each timepoint, except for t = 168 and t = 192 h. An unpaired *t*-test was conducted to determine the statistical significance of the improvement in glucose consumption at each timepoint. Further, two-way ANOVA with a Tukey *post hoc* test was utilized to analyze the differences in glucose uptake between consecutive measuring timepoints and shows significant decreases in glucose uptake from timepoints 24–48 h, 48–72 h, 120–144 h, 144–168 h, and 168–192 h. n = 3.

Measuring the glucose levels in the effluent is a non-invasive readout, therefore other post-trial assays and imaging of the adipocyte samples can occur on the same sample. This comprehensive approach can be used to investigate the long-term insulin response and glucose metabolism of the adipocytes within the perfusion system. The average glucose consumption for the insulin-perfused and non-insulin perfused adipocyte effluents was calculated (raw data in Supplementary Figure S2) and compared during a 10-day trial (Figure 9). Consistent with normal *in situ* physiology, the effluent of the insulin-perfused samples consistently exhibited greater glucose uptake throughout each 24-h period of the 10-day trials compared to the control samples. The results indicated that the increase in glucose uptake in the insulin-perfused adipocytes was statistically significant at every timepoint, with the exception of t = 168 and 192 h (*p* = 0.4405 and 0.1503).

During the 10-day trials, both the insulin-perfused and control groups displayed a consistent decrease in the amount of glucose consumed over time. The highest glucose uptake was observed at t = 24 h for the insulin-perfused adipocytes (10.2 mg/dL) and t = 48 h for the control group (5.3 mg/dL), respectively. This trend continued until t = 240 h, representing the lowest concentration of glucose consumed for both groups (2.8 mg/dL for insulin-perfused adipocytes and 1.2 mg/dL for control). These findings suggest that our microfluidic system successfully recapitulated *in vivo* functionality by demonstrating an increase in glucose uptake in response to insulin perfusion. However, persistent decline in glucose consumption towards the end of the 10-day trial may be indicative of a reduction in adipocyte glucose metabolism or a potential oversaturation of insulin. To further analyze the differences in glucose uptake between consecutive measuring timepoints, a two-way ANOVA with a Tukey *post hoc* test was utilized. The results revealed significant reductions in glucose consumption for the insulin-exposed adipocytes between hours 48–72 h, 96–120 h, and 144–168 h. Similarly, the control group demonstrated significant decreases in glucose uptake from timepoints 24–48 h, 48–72 h, 120–144 h, 144–168 h, and 168–192 h. Overall, this illustrates the significant impact of insulin perfusion on glucose uptake, providing strong evidence of enhanced functionality and reinforces the validity of our microfluidic system for reproducing an *in vivo* physiological drug response and cellular functionality in a 3D *in vitro* environment.

## 4 Conclusion

Ultimately, functionality of mature adipocytes was found to be conserved through insulin-induced elevation of glucose consumption, as a proof of concept for on-chip drug screening models. Unlike static, 2D culturing systems, microphysiological systems provide the capability to test the drug-mediated response of a heterogeneous cell culture, while allowing for temporally dynamic drug perfusion. Among the most significant functions of OOC is the platform’s ability to perform preclinical autologous drug screening, pharmacodynamics, and disease modeling in an organ-specific microenvironment. Due to the prevalence of obesity and its comorbidities globally, the key role of WAT as homeostatic regulator of metabolism and energy storage in the endocrine system, and the lack of knowledge about the pathophysiology behind these processes, WAT has emerged as a drug target with significant therapeutic implications. Therefore, the ability to develop a system for reproducible drug exposure and response for functional WAT is highly relevant. Future experiments will be conducted to explore the capacity of our system as a drug delivery tool and disease modeling tool by inducing alternative drug-induced responses on-chip.

Furthermore, this study validates the use of two non-invasive readout techniques, resazurin and glucose uptake, to obtain continuous data for adipose tissue OOC models. The developed techniques address a critical challenge of accounting for patient variability while creating a physiologically relevant model. The Micronit platform proved to be a reproducible model that can effectively maintain adipocyte viability, metabolic activity, and basic functionality. Within the platform, dynamic adipocyte size remodeling was observed initially to adjust to the hydrogel stiffness as well as adipocyte hypertrophy in response to the high nutrient environment. Insulin-mediated glucose uptake was demonstrated, validating the ability to mimic *in vivo* responses on a Micronit platform. These non-invasive readouts contribute to advancing the understanding of dynamic tissue functions and can provide valuable tools for drug discovery and disease modelling *in vitro*.

## Data Availability

The raw data supporting the conclusions of this article will be made available by the authors, without undue reservation.

## Supplementary material

SUPPLEMENTARY FIGURE S1
**(A–F)** Macroscopic images of the HA hydrogel with varying ratios of Glycosil:Extralink. **(G)** 4:1 hydrogels with and without SVF were imaged, stained with BODIPY (green = lipids), DAPI (blue = nuclei), and Phalloidin 555 (red = actin cytoskeleton). Scale bar = 100 µm.

SUPPLEMENTARY FIGURE S2 Raw data of glucose concentrations measured from effluent media *n* = 3. The control media that was perfused through acellular chips maintained the highest concentration of glucose while the samples that were supplemented with insulin have the lowest concentration of glucose.
