# Supplementary material for: Optimization and validation of a fat-on-a-chip model for non-invasive therapeutic drug discovery
Source: Front Bioeng Biotechnol. 2024 Jun 25;12:1404327. doi: 10.3389/fbioe.2024.1404327 (PMC11235003; doi:10.3389/fbioe.2024.1404327)
Supplement: Supplementary file 1 [file Presentation1.pdf]

Supplementary Material

1 Supplementary Data

1.1 Hydrogel Gel Ratio Optimization

A hyaluronic acid (HA) based hydrogel was used to create a 3D environment within the Micronit platform. HA is an important ECM component in adipose tissue and contributes to ECM remodeling and metabolic regulation (Sun, Li, and Scherer 2023). Preliminary data was conducted to optimize the HA based hydrogel stiffness by varying the ratio of the thiol modified HA (Glycosil) and the thiol reactive crosslinker (Extralink). The ratios of Glycosil to Extralink investigated were 4:1, 20:1, and 40:1 (Supplemental Figure 1 A - F) because recent literature has shown adipose tissue models with these ratios (Julia et al. 2022). The adipocytes in the 20:1 and 40:1 hydrogels are seen to accumulate around the perimeter of the well, leaving a gap in the center of the well, likely because the hydrogel did not have enough crosslinking agent to gel entirely. The second condition tested was the addition of the stromal vascular fraction. 4:1 hydrogels with and without SVF were images to verify the accumulation of SVF (Figure 1 G-H).

1.2 Viability Data

An LDH ELISA readout was performed to measure cell death and further validate the use of resazurin as a marker for viability. Over the course of a 240-hour trial, effluent collected from 20 µL, 30 µL, and 40 µL adipocyte-hystem samples exhibited LDH secretion concentrations below the minimum threshold range of the ELISA kit of 0.056 ng/mL, and therefore, could not be used as an indicator of cytotoxicity. However, this suggests that there is little toxicity.

1.3 Glucose Uptake

Glucose concentration was determined using the GlucCell™ Glucose Monitoring System and a GlucCell™ Glucose Test Strips. The effluent samples were diluted with standard DMEM by a factor of two to be within the optimal glucose monitoring range. Each experiment consisted of 2 chips without insulin, 2 chips with insulin, and 2 control groups with no cells. The raw data (Supplemental Figure 2) was later used to calculate the glucose uptake. As expected, the control media that was perfused through acellular chips maintained the highest concentration of glucose while the samples that were supplemented with insulin had the lowest concentration of glucose.

2 Supplementary Figures and Tables

2.1 Supplementary Figures

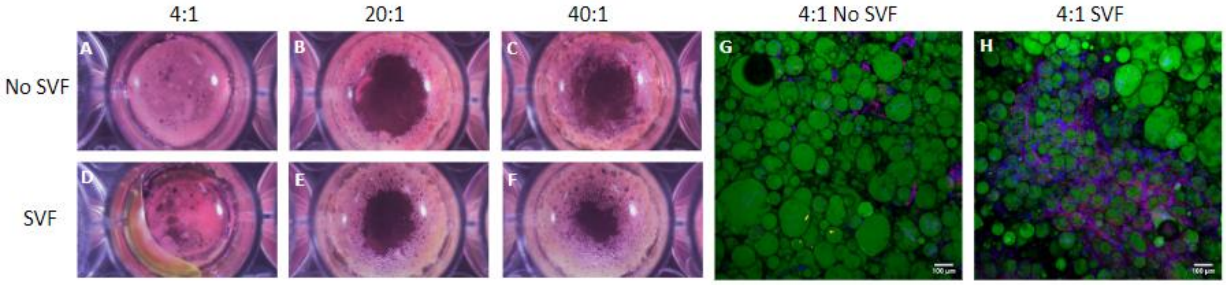

Supplemental Figure 1. (**A-F)** Macroscopic images of the HA hydrogel with varying ratios of Glycosil:Extralink. **(G)** 4:1 hydrogels with and without SVF were imaged, stained with BODIPY (green = lipids), DAPI (blue = nuclei), and Phalloidin 555 (red = actin cytoskeleton). Scale bar = 100 µm.

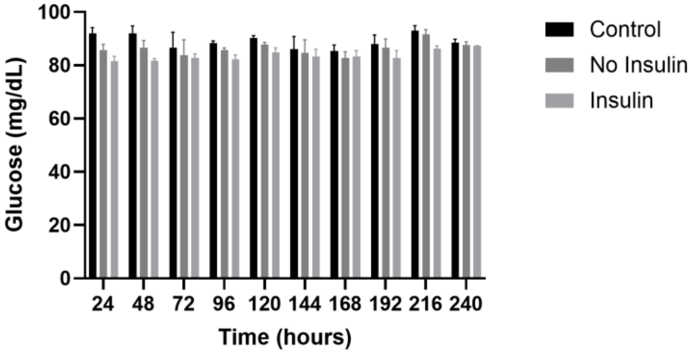

Supplemental Figure 2. Raw data of glucose concentrations measured from effluent media n=3. The control media that was perfused through acellular chips maintained the highest concentration of glucose while the samples that were supplemented with insulin have the lowest concentration of glucose.
